# Comparison of Knowledge of Lactose Intolerance and Cow’s Milk Allergy Among the Medical Students at Two Universities in Saudi Arabia

**DOI:** 10.7759/cureus.50326

**Published:** 2023-12-11

**Authors:** Salah M Bakry, Ziad Banoun, Ammar Abdulfattah, Fawaz Alkhatib, Mussad Almhmadi, Mohammed Alharbi, Adel Alluhaybi, Mohammed O Krenshi, Fahad Alharthi, Samar Ekram

**Affiliations:** 1 Department of Medicine and Surgery, College of Medicine, Umm Al-Qura University, Makkah, SAU; 2 Department of Medicine and Surgery, College of Medicine, King Abdulaziz University, Makkah, SAU; 3 Department of Medical Genetics, College of Medicine, Umm Al-Qura University, Makkah, SAU

**Keywords:** saudi arabia, medical students, knowledge, cow’s milk allergy, lactose intolerance

## Abstract

Lactose intolerance is a condition causing an inability to absorb and digest lactose leading to gastrointestinal symptoms such as abdominal pain, diarrhea, and flatulence. Because of the similarities between lactose intolerance and cow’s milk allergy, it is becoming necessary to increase physicians’ understanding of these two diseases. Consequently, we aimed to determine the level of knowledge of lactose intolerance and cow’s milk allergy among medical students.

An electronic survey was distributed to 399 medical students at two universities in Saudi Arabia from October to November 2022.

The majority of the respondents had an inadequate knowledge of both lactose intolerance and cow’s milk allergy (99.75% and 97.99%, respectively).

According to the study’s results that showed a lack of awareness among health-related students, further studies and awareness programs are highly recommended.

## Introduction

About 70% of the world’s population has lactose intolerance (LI), which is caused by the inability to digest and absorb lactose (the sugar found in milk and other dairy products) [1,2]. Lactose has to be broken down by lactase enzyme into D-glucose and D-galactose before it can be absorbed into the bloodstream [1,2]. After weaning, the expression of the lactase enzyme gradually decreases in most of the world’s population, known as lactase non-persistence [2,3]. Population genomics research suggests that lactase persistence, or the capacity to digest lactose after infancy, first appeared during the Eurasian Bronze Age (3000-1000 BC) [1,4]. The regular consumption of milk and fermented milk products may have improved people’s nutritional condition [1,2]. 

LI is characterized by mild gastrointestinal symptoms such as diarrhea, flatulence, and abdominal pain [5]. Individuals under five can usually tolerate lactose well since primary LI occurs in people over five [5]. LI and cow’s milk allergy (CMA) symptoms in the gastrointestinal tract are so similar that many parents and physicians conflate them [1].

Owing to the similarities of LI and CMA, it is crucial to increase physicians’ awareness and knowledge of both diseases. Limited investigation shows physicians’ inadequate understanding of LI and CMA [6-8]. A recent study reveals that nutritionists have limited knowledge of the nutritional management of LI [9]. By contrast, another study shows that the leading suggestions given by pediatricians and nutritionists for treating CMA are conceptually incorrect [10]. Consequently, this study aims to investigate the level of knowledge of LI and CMA among the medical students at both Umm Al-Qura University and King Abdulaziz University.

## Materials and methods

Study design and participants

In this cross-sectional study, we targeted the medical students of both Umm Al-Qura University and King Abdulaziz University to compare their respective levels of knowledge of LI and CMA from October 10 to November 10, 2022, and we excluded those who refused to participate in the study.

Sample size and ethical consideration

We calculated the sample size using the software of Open Epi [11]. To obtain 95% confidence and a 5% acceptable error margin, we collected the data from 399 medical students using the convenience sampling method.

We obtained ethical approval from the biomedical ethics committee of the College of Medicine at Umm Al-Qura University, Makkah, Saudi Arabia (approval number HAPO-02-K-012-2022-09-1203).

Study procedure and questionnaire design

The study questionnaire was adopted and modified from previously published studies [10,12]. The survey was electronically designed using Google Forms and distributed on social media platforms (Telegram and WhatsApp).

It was divided into three parts. The first part was designed to collect the participants’ demographic data, the second part included questions to assess their knowledge of CMA, and the third part aimed to assess their knowledge of LI. The questionnaire was accompanied by a consent form to be signed by all the respondents before they moved on to the main survey and the email of the corresponding author for any inquiries.

Statistical analysis

The final data were stored in Microsoft Excel spreadsheets to check their completeness and eliminate typographical errors. The descriptive statistics were represented by percentages for the categorical variables and mean and standard deviation for the continuous variables, using a Statistical Package for the Social Studies 23 spreadsheet (IBM, Armonk, NY). Finally, the categorical variables were computed using the independent chi-square test to determine all the knowledge-related factors. A p-value less than or equal to 5% was considered significant.

## Results

This survey-based study targeted medical students at two universities in Saudi Arabia. We enrolled 399 students with a mean age of 21.38 (SD=1.89). Most of the students were men (n=224, 56.1%). Most of the responses were from the students at Umm Al-Qura University (n=308, 77.2%), with fewer responses from the students from King Abdulaziz University (n=91, 22.8%). Furthermore, most of the responses were from third-year medical students (n=111, 27.8%), followed by sixth-year students (n=108, 27.1%) (Table 1).

**Table 1:** Participants’ demography

Categories		N	%
Age groups	17-20	146	36.6
	21-24	236	59.1
	25-27	17	4.3
Gender	Male	224	56.1
	Female	175	43.9
University	Umm Al-Qura University	308	77.2
	King Abdulaziz University	91	22.8
Academic year	Premedical year	25	6.3
	2nd year	38	9.5
	3rd year	111	27.8
	4th year	38	9.5
	5th year	79	19.8
	6th year	108	27.1
History of cow's milk allergy	Yes	22	5.5
	No	377	94.5
History of lactose intolerance	Yes	52	13.0
	No	347	87.0
Family history of cow's milk allergy	Yes	26	6.5
	No	373	93.5
Family history of lactose intolerance	Yes	72	18.0
	No	327	82.0
Ever heard about cow's milk allergy	Yes	230	57.6
	No	169	42.4
Ever heard about lactose intolerance	Yes	350	87.7
	No	49	12.3
Age (mean) (standard deviation)	(Mean=21.38) (SD=1.89)		

Most of the students had a negative past history of both CMA and LI (94.5% and 87%, respectively). By contrast, 5.5% of the students had a history of CMA, while 13% had a history of LI. Moreover, 6.5% had a positive family history of CMA compared with 18% for LI (Table 1).

Most of the students had a good level of awareness of CMA and LI [(n=230, 57.6%) and (n=350, 87.7%), respectively] (Table 2).

**Table 2:** The association between participants’ demography and knowledge score of cow's milk allergy and lactose intolerance

Categories		Knowledge score of cow milk allergy		P-Value	Knowledge score of lactose intolerance		P-Value
		Poor level of knowledge	Good level of knowledge		Poor level of knowledge	Good level of knowledge	
Age groups	17-20	142	4	0.649	146	0	0.707
	21-24	232	4		235	1	
	25-27	17	0		17	0	
Gender	Male	222	2	0.073	223	1	0.376
	Female	169	6		175	0	
University	Umm Al-Qura University	302	6	0.881	308	0	0.065
	King Abdulaziz University	89	2		90	1	
Academic year	Premedical year	23	2	0.107	25	0	0.541
	2nd year	36	2		38	0	
	3rd year	109	2		111	0	
	4th year	38	0		38	0	
	5th year	79	0		78	1	
	6th year	106	2		108	0	
History of cow's milk allergy	Yes	21	1	0.382	22	0	0.809
	No	370	7		376	1	
History of lactose intolerance	Yes	51	1	0.964	52	0	0.698
	No	340	7		346	1	
Family history of cow's milk allergy	Yes	26	0	0.451	26	0	0.792
	No	365	8		372	1	
Family history of lactose intolerance	Yes	72	0	0.180	72	0	0.638
	No	319	8		326	1	
Ever heard about cow's milk allergy	Yes	224	6	0.316	229	1	0.391
	No	167	2		169	0	
Ever heard about lactose intolerance	Yes	343	7	0.985	349	1	0.708
	No	48	1		49	0	

**Figure 1:**
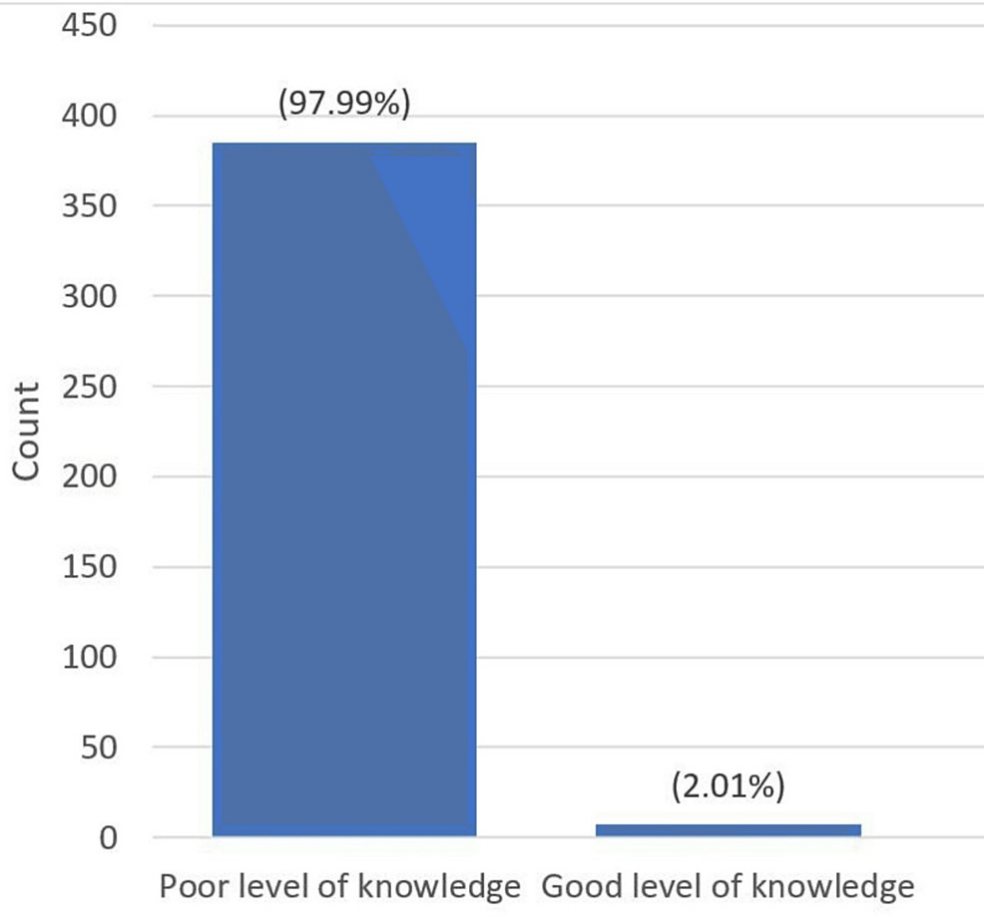
Knowledge score of CMA CMA, cow's milk allergy.

**Figure 2:**
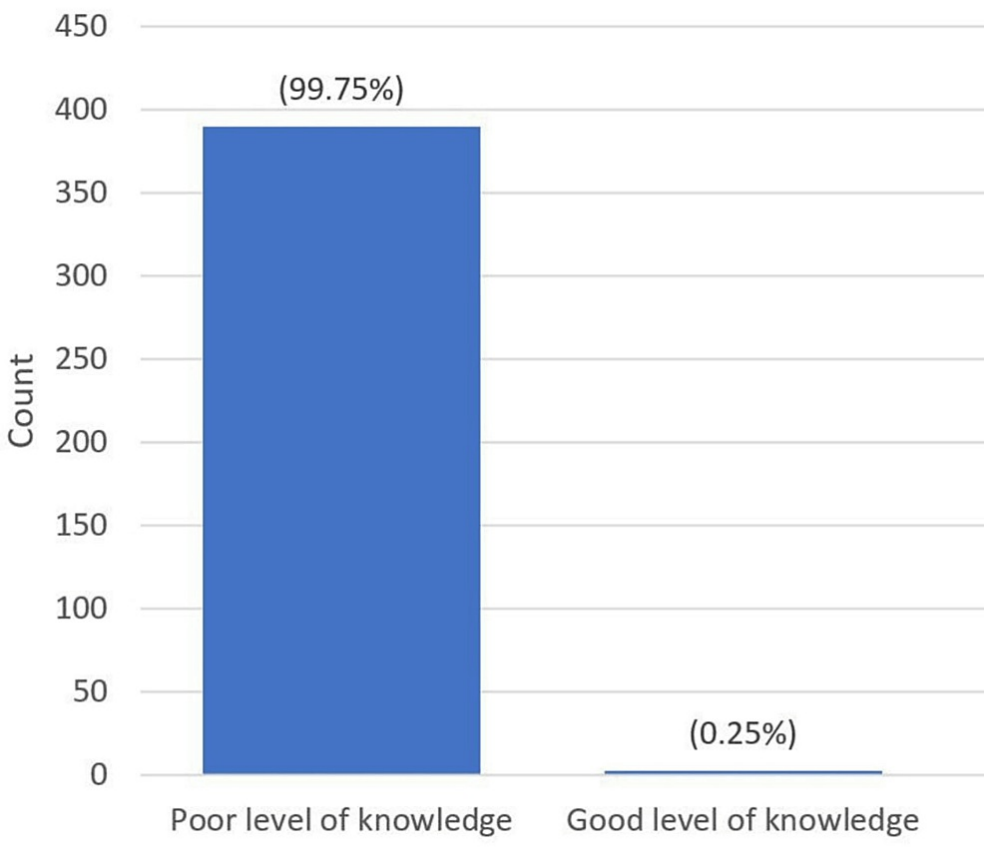
Knowledge score of LI LI, lactose intolerance.

However, this was far different from the knowledge scores of both CMA and LI, with the majority of the students having inadequate knowledge of both CMA and LI (97.99% and 99.75%, respectively) (Figures 1, 2).

## Discussion

To our knowledge, this is the first study that estimates the level of understanding of LI and CMA among medical students. Most studies have been conducted among physicians, and the level of knowledge about each disease was investigated separately [9,12].

Knowledge of LI

LI is a gastrointestinal disorder encountered among more than 65% of the human population [13,14]. LI is characterized by the incapability to absorb the lactose sugar in dairy products and milk, which leads to stomach pain or discomfort [13,14]. So, it is vital to improve public awareness of LI with the main knowledge about symptoms and the use of a lactose-free diet with which LI can be significantly decreased. 

LI is not life-threatening, but it does affect patients’ quality of life and well-being, and they may experience extreme discomfort caused by ineffective treatment [12,15]. Thus, medical students must recognize its diagnosis workup and management. 

According to our findings, most participants had poor knowledge of LI. Similarly, two different studies conducted in Saudi Arabia among nutritionists and the general population showed a limited level of knowledge regarding LI [9,13]. However, these disagree with a study conducted among clinicians, which found that they understood the general aspects of LI but not the diagnostic aspects [12].

Knowledge of CMA

Owing to the early representation of CMA symptoms during childhood, a study has suggested that it is essential for primary care physicians to be aware of the circumstances and prepared to make a correct diagnosis and use current treatment and management approaches [16].

Our study reports that most students have poor knowledge of CMA. Another study of food allergies that involved 407 pediatricians and primary care doctors demonstrated that the basic knowledge of clinicians was only average; however, they were comfortable following up with children who had a food allergy [17]. Another study showed that pediatricians receive insufficient training in diagnosing, treating, and managing anaphylaxis caused by food allergies [18]. Furthermore, the availability of insufficient information on the treatment and diagnosis of CMA and the use of adrenaline autoinjectors was found in a study of 126 family doctors practicing in the Turkish province of Malatya [16-19].

This study carries some possible limitations. First, as this is a cross-sectional survey-based research, selective bias could be a limitation. The sample size could be increased to overcome this. Furthermore, this study’s findings cannot be generalized to all Saudi universities; therefore, we recommend further studies among all Saudi universities.

## Conclusions

This study shows that most students have an inadequate understanding of CMA and LI in contrast to the findings of international studies. Therefore, we recommend more awareness programs and further studies among health-related students and clinicians.
